# Effectiveness of the Treatment of Physiotherapy in the Congenital Muscular Torticollis: A Systematic Review

**DOI:** 10.3390/children11010008

**Published:** 2023-12-20

**Authors:** Manuel Rodríguez-Huguet, Daniel Rodríguez-Almagro, Miguel Ángel Rosety-Rodríguez, Maria Jesus Vinolo-Gil, Carmen Ayala-Martínez, Jorge Góngora-Rodríguez

**Affiliations:** 1 Department of Nursing and Physiotherapy, University of Cádiz, 11009 Cádiz, Spain; mariajesus.vinolo@uca.es (M.J.V.-G.); jorge.gongora@uca.es (J.G.-R.); 2 Department of Nursing, Physical Therapy and Medicine, University of Almería, 04120 Almería, Spain; dra243@ual.es; 3 Move-It Research Group, Biomedical Research and Innovation Institute of Cádiz (INiBICA), Puerta del Mar University Hospital, University of Cádiz, Plaza Fragela, s/n, 11003 Cádiz, Spain; miguelangel.rosety@uca.es; 4 Rehabilitation Clinical Management Unit, Interlevels-Intercenters Hospital Puerta del Mar, Hospital Puerto Real, Cádiz Bay-La Janda Health District, 11006 Cádiz, Spain; 5 Research Unit, Biomedical Research and Innovation Institute of Cádiz (INiBICA), Puerta del Mar University Hospital, University of Cádiz, 11009 Cádiz, Spain; 6 Doctoral School, University of Cádiz, 11003 Cádiz, Spain; carmenmaria.ayamar@alum.uca.es

**Keywords:** congenital muscular torticollis, exercise, pediatric telemonitoring, physical therapy, rehabilitation

## Abstract

A single congenital muscular torticollis (CMT) is a postural musculoskeletal deformity and is characterized by the shortening or stiffness of the sternocleidomastoid muscle. The reported incidence of CMT ranges from 0.2% to 2%. The objective is to evaluate the effect of physical therapy programs on CMT. For the search, PubMed, Scopus, Web of Science, PEDro and Cochrane databases were used. Randomized controlled trials published between 2018 and 2023 have been included. This study follows the PRISMA 2020 statement and has been registered in the PROSPERO database. Finally, six studies were included. The cervical range of motion (ROM) in rotation was the most analyzed variable, followed by the ultrasound evaluation; one of the studies included the analysis of children’s motor development with the Alberta scale. All research found benefits associated with soft tissue mobilization, passive stretching techniques and manual therapy of the cervical spine. In conclusion, it is possible to recommend manual therapy and passive stretching techniques for the treatment of CMT, with significant results on the cervical ROM.

## 1. Introduction

Congenital muscular torticollis (CMT) is musculoskeletal disorder (“tortum collum” or wry neck) characterized by a neck deformity that occurs in newborns or during the first months of life [1,2,3]. This deformity is due to a unilateral shortening of the sternocleidomastoid muscle [4,5], which can be caused by the intrauterine position of the head, an intrauterine vascular alteration of the muscle, or obstetric trauma [3] (although the prenatal ischemic episode seems to stand out above the obstetric trauma) [4]. CMT is manifested by a tilt of the neck towards the injured side (ipsilateral latero-flexion) and the head turning in the opposite direction (contralateral rotation) [2,4,6].

In some cases, a small lump may be observed on the lateral side of the neck, due to muscle fibrosis [1]. In addition, other muscles such as the splenius, the trapezius and the scalene muscles may also be affected [3,4]. Therefore, CMT is a pathology that affects the mobility of the neck of a newborn [1,5,7,8]; likewise, it can be associated with craniofacial deformity and asymmetry [7,8]. 

This disorder is the third of the pathologies that most affects the functionality of the children, behind congenital hip dysplasia and congenital clubfoot [4]. Specifically, the reported incidence of CMT ranges from 0.2% to 2% [2,4,9]. It can be considered more frequent in primiparous and with a certain predominance of the right side [4].

Sometimes the disorder can resolve spontaneously [4]. However, physiotherapy treatments could play a fundamental role in restoring functionality and promoting motor development in children [4,5,7]. Thus, the common objective of the different therapeutic interventions for CMT is to increase the extensibility and strength of the sternocleidomastoid muscle [5,7]. In this way, it is possible to prevent secondary alterations such as body asymmetries compensating for lack of movement [7], taking into account that it is appropriate for children to achieve full range of motion (ROM), which is 100° or more in rotation and 65° or more in lateral flexion [10].

The objective of this systematic review is to evaluate the effect of physical therapy programs on CMT.

## 2. Materials and Methods

### 2.1. Review Protocol and Search Strategy

The entire protocol and search strategy for this systematic review were conducted following the guidelines of Preferred Reporting for Systematic Reviews and Meta-Analyses (PRISMA2020) statement. Additionally, the study information was registered with the International Prospective Register of Systematic Reviews (PROSPERO). The databases used for the review were the following: Cochrane Library, PEDro, Pubmed, SCOPUS and Web of Science. The review of articles was carried out between September and October 2023.

The search strategy was established using the PICO question format: in children with CMT (P = population), are physical therapy modalities (I = intervention) compared to no intervention or medical treatments (C = comparison); effective to improve mobility or functionality (O = outcomes)? The final search formula was defined by using structured language descriptors associated by the Boolean operator “AND”; accordingly, the formula entered was “congenital muscular torticollis AND physical therapy” in Cochrane Library, Pubmed, SCOPUS and Web of Science, and exclusively “congenital muscular torticollis” in PEDro, because this database is already focused on evidence in physiotherapy and limits the use of Boolean operators.

### 2.2. Eligibility Criteria, Study Selection and Data Collection Process

The search and identification of studies was carried out by two blinded evaluators independently, with a third evaluator in charge of resolving the discrepancies. The selection of articles was limited to randomized clinical trials published between 2018 and 2023. Studies had to focus on physical therapy treatment in children with CMT. Likewise, the articles had to include the analysis of the cervical ROM as one of their main variables. And only articles published in English or Spanish were considered. 

Duplicate references were eliminated, and a screening was developed based on the reading of the title and abstract. All articles focused on other pathologies that did not meet the inclusion criteria or scored less than 6 on the PEDro scale were excluded.

The total number of the sample and each group was analyzed for each study; the variables, characteristics of each treatment, duration of the intervention, follow-up time and the results of each trial were recorded.

### 2.3. Quality Assessment of Studies

The methodological quality of the articles was analyzed using the PEDro scale. This scale offers high reliability, it is an appropriate tool to assess the quality of randomized clinical trials [11]. The scale has 11 items, although finally it offers a score from 0 to 10. Only articles of good quality were selected (minimum score of 6), and the score was included in the analysis of the results. 

The PEDro scale includes items that allow us to analyze selection bias (items 2 and 3), performance bias (items 5 and 6) and detection bias (item 7). Therefore, it is possible to consider that a high score on the PEDro scale represents high methodological quality and zero risk of bias; on the contrary, a low score could be related to a high risk of bias and poor methodological quality.

## 3. Results

### 3.1. Study Selection

**Figure 1:**
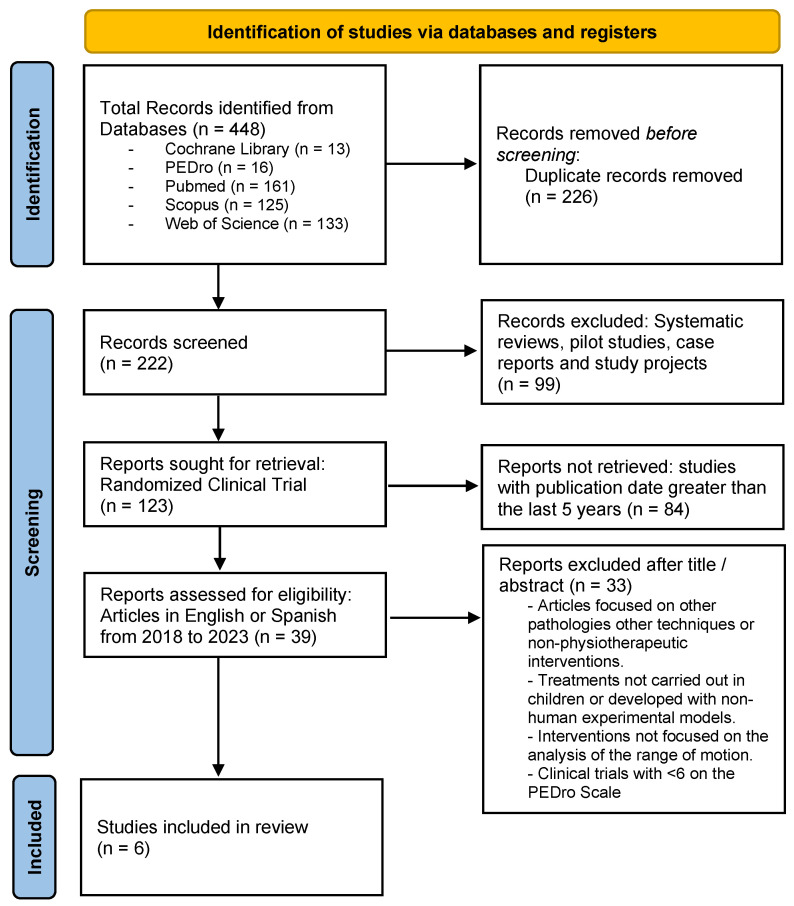
PRISMA flow diagram. Identification of the results obtained from the databases.

The initial search strategy in the indicated databases offered a total of 448 results, of which six were selected after applying the inclusion and exclusion criteria. The selection process is indicated in the PRISMA flow diagram (Figure 1).

### 3.2. Sample Population Characteristics and Methodological Quality Assessment

The total sample size of the set of studies analyzed was composed of 326 individuals. All studies assessed changes in the cervical ROM in children under one year of age with CMT. Specifically, most studies selected children less than 3 months old [12,13,14]. On the other hand, Keklicek et al. (2018) [15] and Pastor-Pons et al. (2021) [16] included children under 6 months and 28 weeks, respectively. And the investigation of Durguti et al. (2019) [17] selected children between 1 and 9 months, establishing follow-up based on age group. The sex of the participants was recorded (in total 165 girls and 161 boys). In addition, the lateral predominance of the involvement and the type of delivery were recorded in some of the clinical trials.

The methodological quality of the articles was assessed by the PEDro scale; Table 1 includes the score obtained by each trial and characteristics of these. All reviewed research obtained a score above 6, which establishes a good methodological quality, and three of the articles had a confirmed score of 7 points on the scale. In addition, Appendix A contains detailed scoring information for each item. Regarding possible biases, almost all research does not obtain a score in items 3, 5 and 6. It is possible to highlight the appearance of a bias of overestimation of the results among the risks of biases detected in the selected articles because in none of the studies analyzed was blinding of patients or therapists carried out, although this is a common limitation of many physiotherapy interventions. 

### 3.3. Outcomes Measurements and Assessment Time

Cervical ROM was the most analyzed study variable and had the greatest impact on CMT dysfunction [12,13,14,15,16,17], which includes the assessment of rotation [12,13,14,15,16,17] and lateral flexion [12,15,17] movements. Also, head tilt was measured with photographic methods [15]. 

The thickness of the sternocleidomastoid muscle was the second most analyzed variable, based on an ultrasound evaluation [12,13,14]. In the same way, Hwang et al. (2019) incorporates shear-wave velocity to assess tissue stiffness [12] and the assessment of the thickness ratio between the affected side and the contralateral side is included in one study [14]. Furthermore, certain studies included the specific assessment of motor function with the Muscle Function Scale (MFS) [15], and the analysis of psychomotor development with the Alberta Infant Motor Scale (AIMS) [16].

Regarding the assessment time, most of the selected articles develop a short-term follow-up, with baseline and post-assessment [12,13,14,16,17]. Only the study of Keklicek et al. (2018) [15] includes follow-up at 6, 12, and 18 weeks; and the investigation of Kwon et al. (2023) [13] incorporates the assessment of ROM in a month later treatment in group 1, and immediately after the last treatment in groups 2 and 3, and thickness was measured 3 months post-treatment.

### 3.4. Intervention Protocols and Effects of Treatments

In general, the treatments were developed by physiotherapists specializing in manual therapy in the pediatric care, and these interventions were based above all on the application of massage and passive stretching.

Passive stretching techniques were focused directly on stretching the sternocleidomastoid muscle [12,13,14,15,17]; the protocol of Durguti et al. (2019) [17] includes stretching held for 10 to 30 s, performing 10 repetitions in each session after the massage treatment to increase blood flow (this treatment was followed in the three groups, analyzing the changes depending on the age of the patients). Other studies describe protocol stretching based on three sets of 15 repetitions of 10 s with 5–10 s of rest [13]; stretching exercise in periods of 30 s with 10 s of rest [15]; and sternocleidomastoid muscle elongation and strengthening of the contralateral side through position changes and stimuli to induce movement or 10 to 20 repetitions of 10 s stretches, with one minute of rest [14]. 

Massage maneuvers or soft tissue mobilization were based on rhythmic muscle mobilization techniques with a pincer grip and associated with rotation movement [15], effleurage techniques [17], or manual therapy to mobilize the occiput, atlas and axis, specifically, myofascial induction techniques to relax the cervical myofascial structures, applying gentle traction and movement assistance [16].

Instrumental interventions with electrotherapy devices were focused on the application of ultrasound, application times ranged from 5 [12,13] to 30 min [14], and the intensity was 0.5 to 5 W/cm^2^. Also, the application of microcurrent therapy was provided for 30 min 3 times a week, with an intensity of 200 µA [13]. In addition, the investigations of Keklicek et al. (2018) [15], Kwon et al. (2023) [13] and Pastor-Pons et al. (2021) [16] include a home stretching program and an education program for caregivers, and Hwang et al. (2019) [12] also reflects these recommendations for parents.

Therefore, the comparison was developed between the duration of the same treatment protocol or between the combination of techniques together with the isolated application of the techniques or the treatment with exercise guidelines at home. 

Regarding the total duration of the treatment and the time of each session, it is possible to highlight that, in the study of Hwang et al. (2019) [12], patients received a 30 min treatment per week for 3 months, Pastor-Pons et al. (2021) [16] does one session per week for 10 weeks, other studies concentrate the treatment in several sessions per week between 1 and 12 weeks [13,15,17] and the investigation of Song et al. (2021) [14] extend treatment until head tilt is reduced.

The effects obtained can be considered beneficial for patients. However, it would be appropriate to highlight certain conditions. Keklicek et al. (2018) [15] obtained statistically significant differences on head tilt positioning and rotation ROM (*p* = 0.001) in the intergroup analysis at intermediate follow-up at 6 weeks. But in the follow-up at the end of the intervention, both groups (soft tissue mobilization and stretching program and recommendations at home or exclusively stretching and recommendations at home) obtained positive results with no differences between them.

Likewise, in the investigation of Pastor-Pons et al. (2021) [16], the patients who participated in the intervention group with manual therapy and educational program for caregivers presented significant changes in rotation ROM (*p* = 0.001) compared to the group that received only the care program. However, in these articles, no differences were observed between groups in the MFS or the AIMS [15,16].

Song et al. (2021) [14] showed that the group with treatment of postural control exercises and passive stretching therapy achieves a significantly higher increase in rotation ROM (*p* < 0.05) than the group with active-assist movement and the group with US treatment. On the contrary, no significant differences were observed between groups on sternocleidomastoid muscle thickening and ratio of the sternocleidomastoid muscle thickness on the affected side to the non-affected side.

In the other studies, it was possible to find statistically significant differences in the pre-treatment and post-treatment comparison (*p* < 0.001) that justify the inclusion of the treatment [12,17]. Changes in ROM and thickness are observed in the intragroup analysis (*p* < 0.05) and greater differences in the intensive treatment groups (*p* < 0.05) [13]. 

## 4. Discussion

Current evidence reflects that CMT is a relevant musculoskeletal alteration, which could limit functionality and condition the development of the infant [18]. The origin of this postural deformity seems to be in the sternocleidomastoid muscle; therefore, the objective of the treatments is to act on that muscle and correct the position [3,10,18]. This explains why the ROM is the main variable analyzed in this review, thus all the articles study the changes in the rotation ROM and in the tilted position of the head or the lateral-flexion ROM [12,13,14,15,16,17]. Furthermore, the thickness of the sternocleidomastoid muscle will be a diagnostic and predictive factor [19], on which treatment could also act with positive results to reduce fibrosis and to increase mobilization of the tissue [12,13].

The results obtained in the articles analyzed suggest that there are benefits when applying physical therapy treatment on CMT, although it is necessary to indicate some considerations. One of the key aspects for the success of the intervention could be the importance of an early approach [17,18]. The most notable differences appear when the treatment is developed early [17], intensively [12], and the parents are involved [13,15,16]. The training of caregivers allows consistency of treatment, especially as it favors adherence to treatment thanks to position changes and repetition of stretches. Furthermore, in future research, it could be interesting to assess parents’ satisfaction in playing an active role in the treatment, because the bond between caregiver and infant could reinforce the intervention. Otherwise, early attention to the pathology could prevent complications, and thus avoid more complex treatments of a surgical nature or under anesthesia [1,18,20].

In the same way, manual therapy treatment appears to offer beneficial results when focusing on mobilization of the upper cervical region and correction of cranial deformities [8,21], this circumstance stands out in research focused on children with plagiocephaly that appears associated with CMT [16], taking into account that CMT could lead to secondary alterations such as facial asymmetry, scoliosis and other alterations of the spine [7,22]. Also, some studies suggest that limitation in the neck can lead to pelvic dysfunction due to changes in posture and ability to move [9]. 

Therefore, with respect to specific treatment guidelines, it seems appropriate to point out the combination of stretching techniques with the home education program [13,15,16]. The results studied suggest that stretching should be carried out progressively, and maintained between 10 and 30 s, with appropriate rest pauses of around 10 s, in sets of 10 to 20 repetitions [13,14,15,17]. And the treatment could be extended for several weeks, until the position of the head is corrected and the ROM increased [14]. 

Within the treatment, the action of ultrasound seems limited, at least as an isolated treatment; perhaps it could serve as a complement [12,13]. On the other hand, the use of electrotherapy currents in infants can generate controversy, but it can have a place as a complement in the treatment, achieving positive results [13]. There are other studies that agree on the benefits of using microcurrents in patients with CMT [6,23].

It is also notable that the results found on the AIMS scale [16] seem to confirm that CMT does not cause delay in psychomotor development [7].

Finally, it would be advisable to highlight the importance of establishing a global assessment, follow-up and treatment strategy for infants with CMT [24]. Thus, local treatment on the sternocleidomastoid and cervical ROM can be accompanied by active-assisted exercise maneuvers [14] and manual therapy techniques or global massage [25], or other complementary techniques, such as kinesiotaping [24,26].

The strengths of this review are to include physical therapy interventions for CMT based on different methodologies. In this way, a global assessment of the treatment possibilities has been developed combining manual therapy, active mobilization stimuli, electrotherapy and recommendations for caregivers. Also, the search was limited to the last 5 years to assess the most current evidence, so that it was possible to analyze the most recent treatments and their effects. On the other hand, the methodological differences between the studies analyzed could condition the results and could be considered one of the weaknesses of the research. The analysis of the results could also be affected by the risk of bias of each of the investigations, understanding the limitations of blinding depending on the type of interventions and the characteristics of these patients. Likewise, it would be advisable to develop a more in-depth study based on the execution of a meta-analysis; however, this circumstance is conditioned by the characteristics of each article, and the heterogeneity of treatments and the differences in follow-up make it difficult to perform a meta-analysis. Future research could focus on longer-term follow-up, or on the assessment of complications associated with CMT. Soon, it would be convenient to develop experimental studies minimizing the possible biases of the intervention, and a statistical comparison between said results.

## 5. Conclusions

In conclusion, it is possible to recommend manual therapy and passive stretching techniques for the treatment of CMT, with significant results on the cervical ROM. Based on this systematic review, physical therapy plays an important role in the recovery of children with CMT; for this, it is advisable to involve caregivers in an exercise program at home. It is appropriate to establish a correct identification of the CMT and perform treatment on the pathology to enhance children’s motor development and prevent secondary complications.

## Figures and Tables

**Table 1:** Characteristics, interventions and results of the trials included in the systematic review.

Author (Year)	Participants	Outcomes	Assessment Time	Treatment	Results
Durguti et al. (2019) [17] PEDro Score = 6/10	N = 130	ROM	Baseline Post-treatment	15 sessions in 3 weeks: 30′ massage and passive stretching	↑ ROM
Hwang et al. (2019) [12] PEDro Score = 6/10	N = 22	SWV SCM thickness ROM	Baseline Post-treatment	30′ per week for 3 months: 5′ US + massage and passive stretching	↑ SWV ↓ SCM thickness ↑ ROM
Keklicek et al. (2018) [15] PEDro Score = 7/10 *	N = 29 (14/15)	MFS ROM Head tilt	Baseline 6 weeks 12 weeks 18 weeks	Home program of stretching and strengthening exercises + STM (3 times a week for 12 weeks)	↑ MFS ↑ ROM ↑ Head tilt
Kwon et al. (2023) [13] PEDro Score = 6/10	N = 54 (10/29/15)	ROM SCM thickness	Baseline Post-treatment	5′ US + 20′ MT + 30′ MCT: 12 sessions (4 weeks) or 10 sessions (1 week) or 20 sessions (2 weeks)	↑ ROM ↓ SCM thickness
Pastor-Pons et al. (2021) [16] PEDro Score = 7/10 *	N = 34 (17/17)	ROM AIMS	Baseline Post-treatment	Educational program for caregivers and 10 sessions, once a week: 20′ manual therapy	↑ ROM =AIMS
Song et al. (2021) [14] PEDro Score = 7/10 *	N = 57 (19/21/17)	ROM SCM thickness A/N ratio	Baseline Post-treatment	3 times a week for 30′ until the head tilt was ≤ 5 degrees: active-assist movement or passive stretching or US	↑ ROM =SCM thickness =A/N ratio

Abbreviations. * Score confirmed in PEDro webpage; AIMS: Alberta Infant Motor Scale; A/N ratio: ratio of the sternocleidomastoid muscle thickness on the affected side to the non-affected side; MCT: microcurrent therapy; MFS: Muscle Function Scale; MT: manual therapy; ROM: Range of motion; SCM: sternocleidomastoid muscle; STM: Soft tissue mobilization; SWV: Shear-wave velocity; US: therapeutic ultrasound; ↑: increase; ↓: decrease.

## Data Availability

No new data were created or analyzed in this study. Data sharing is not applicable to this article.

## Appendix A

The Appendix A (Table A1) shows the score of each clinical trial for each item of the PEDro scale. Specific questions are included within the assessment items of the PEDro scale to provide information on the eligibility criteria, randomization, allocation methodology, equity and similarity between groups at the beginning of treatment, conditions of blinding of patients, evaluators and therapists, especially for those evaluators who recorded key variables, follow-up of at least 85% of study subjects, application of the treatment assigned to each group, statistical comparison information between groups, and measurements and variability of results for at least one key outcome.

**Table A1:** PEDro Score.

Author (Year)		PEDro Scale										
	1	2	3	4	5	6	7	8	9	10	11	Score
Durguti et al. (2019) [17]	Y	N	N	Y	N	N	Y	Y	Y	Y	Y	6/10
Hwang et al. (2019) [12]	Y	N	N	Y	N	N	Y	Y	Y	Y	Y	6/10
Keklicek et al. (2018) [15]	Y	Y	Y	Y	N	N	N	Y	Y	Y	Y	7/10 *
Kwon et al. (2023) [13]	Y	N	N	Y	N	N	Y	Y	Y	Y	Y	6/10
Pastor-Pons et al. (2021) [16]	Y	Y	N	Y	N	N	Y	Y	Y	Y	Y	7/10 *
Song et al. (2021) [14]	Y	Y	N	Y	N	N	Y	Y	Y	Y	Y	7/10 *

Abbreviations. * Score confirmed in PEDro webpage; N: no; Y: Yes. Details of items. 1: Eligibility criteria; 2: Random allocation; 3: Concealed allocation; 4: Baseline comparability; 5: Blind subjects; 6: Blind therapists; 7: Blind assessors; 8: Adequate follow-up; 9: Intention-to-treat analysis; 10: Between-group comparisons; 11: Point estimates and variability. Note: Eligibility criteria (item 1) does not contribute to total score.
